# Long-lived association between Avalonia and the Meguma terrane deduced from zircon geochronology of metasedimentary granulites

**DOI:** 10.1038/s41598-019-40673-9

**Published:** 2019-03-11

**Authors:** J. Gregory Shellnutt, J. Victor Owen, Meng-Wan Yeh, Jaroslav Dostal, Dieu T. Nguyen

**Affiliations:** 1 National Taiwan Normal University, Department of Earth Sciences, 88 Tingzhou Road Section 4, Taipei, 11677 Taiwan; 2 Saint Mary’s University, Department of Geology, 923 Robie Street, Halifax, NS B3H 3C3 Canada; 3 Center for General Education, National Taiwan Normal University, 162 Heping East Road Section 1, Taipei, 106 Taiwan

## Abstract

The Acadian Orogeny of the Northern Appalachians was caused by accretion of the peri-Gondwanan terranes Avalonia and Meguma to the eastern margin of Laurentia during the Devonian. The lithotectonic relationship between Avalonia and Meguma prior to accretion is uncertain. Radioisotopic dating of detrital zircons from metasedimentary granulite xenoliths from the structural basement to the Meguma terrane indicates that Avalonia and Meguma were proximal and likely contiguous as they transited the Rheic Ocean. The zircon ages range from the Cryogenian to Late Silurian with a minor Paleoproterozoic peak. Mesoproterozoic zircons are also identified and, coupled with the Ordovician to Silurian zircons, distinguish the rocks from those of the Meguma terrane. Furthermore, three distinct metamorphic events are identified at 399.0 ± 2.1 Ma, 376.9 ± 1.6 Ma, and 353.8 ± 3.3 Ma. We conclude that the granulite facies metamorphism experienced by the metasedimentary rocks occurred 10 to 20 million years after deposition of their protoliths during the initial stages of the Acadian Orogeny whereas the younger events are related to syn- and post-collisional episodes. The implication is that Avalonia and the Meguma terrane jointly transited from Gondwana.

## Introduction

**Figure 1:**
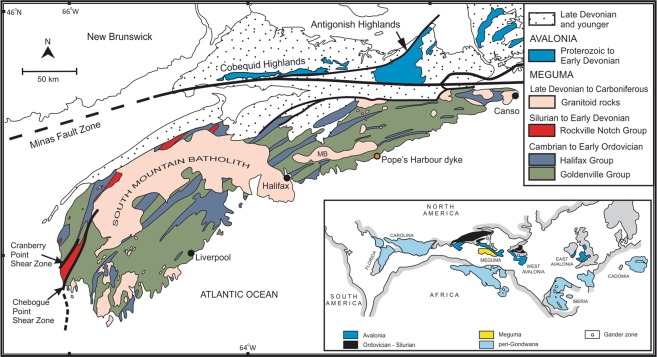
Simplified geological map of mainland Nova Scotia and western Cape Breton showing the distribution of Late Devonian and earlier rock units of Avalonia and Meguma and the location of the Pope’s Harbour dyke. MB = Musquodoboit Batholith. Inset shows the Early Mesozoic reconstruction of Pangea and the locations of Meguma, Avalonia, and other peri-Gondwana terranes.

The Middle Paleozoic Acadian Orogeny of the Appalachians was largely confined to the current northeastern margin of North America^1^. The orogeny was the consequence of the accretion of Avalonia and Meguma, two exotic peri-Gondwanan terranes, and possibly a mantle plume^2–6^. The Avalon terrane is comprised primarily of Neoproterozoic continental margin magmatic rocks and by Cambrian fossil-rich sedimentary rocks. In contrast, the Meguma terrane is composed mainly of flyshoid metasedimentary rocks of the Meguma Supergroup, which includes Cambrian to earliest Ordovician Goldenville Group greywacke and shales and the Lower Ordovician Halifax Group shale^2^. The Meguma Supergroup is >10 km thick and is overlain by the middle Ordovician to early Devonian Rockville Notch Group that is comprised of subaerial to shallow marine sediments (mainly quartzite and shale) and bimodal rift-related volcanic rocks. Subsequent to accretion, a number of Middle Devonian to Early Carboniferous silicic plutons were emplaced across both terranes^7,8^. Avalonia and Meguma are juxtaposed in northern mainland Nova Scotia along the Carboniferous Minas fault (Fig. 1)^9^. The fault likely extends west into the Bay of Fundy and east to the continental shelf.

Avalonia and Meguma have distinct geological and geochemical features and appear to be structurally unrelated terranes^7,10^ but their lithotectonic relationship prior to accretion is unknown^11–13^. Consequently, the nature of the relationship between Avalonia and Meguma is one of the most debated issues of the Northern Appalachians^14^. There are two principal models for the pre-accretion lithotectonic relationship between Avalonia and Meguma. On one hand it is suggested that they evolved independently but rifted from Gondwana and jointly transited the Rheic Ocean before accretion. On the other hand, they are considered to be spatially unrelated and transited at separate and distinct intervals^2,11–13,15^. Constraining the pre-accretion relationship between the two terranes is problematic due to the fact that the Meguma terrane is primary comprised of sedimentary rocks but was thrust over Avalonia and may cover a sizeable portion of it^16^.

Granulite-facies xenoliths are present within a Late Devonian mafic dyke that intrudes the Meguma terrane. The xenoliths are noteworthy because they evidently represent fragments of the structural basement to the Meguma terrane^16–18^. The metaigneous and metasedimentary granulites are entrained as xenoliths in a ~370 million year old, ~15 m wide lamprophyre dyke at Pope’s Harbour (Fig. 1)^19^. The Pope’s Harbour dyke (PHD) is located ~75 km south of the Minas fault zone and is a member of a suite of NW-striking mafic dykes that cut the Meguma Supergroup on the eastern shore of Nova Scotia. The geochemistry of the dykes shows that they are shoshonitic^20^. The dykes are variably evolved, but many samples have primitive compositions with a high *mg* number [>0.70, where *mg*-number = Mg/(Mg + 0·9 × total Fe) atomic], elevated MgO and Ni concentrations (>10 wt.% and 150 ppm, respectively), and low heavy REE concentrations. The xenoliths include aluminous (“metapelitic”, *sensu lato*) and orthopyroxene ± garnet-bearing tonalitic gneisses, and dioritic to ultramafic rocks^17^. The metapelites are most abundant but some were disaggregated and it is common to observe xenocrysts of garnet and aluminosilicate (silliminatized kyanite and sillimanite) minerals in the dyke.

Isotopic evidence from both the metaigneous and metasedimentary xenoliths indicates they are distinct from the overlying Meguma terrane^16^. The metaigneous granulites have chondritic to moderately radiogenic Sr and Nd values (^87^Sr/^86^Sr_i_ = 0.70285 to 0.70500; ε_Nd_(*t*) = −2.03 to +5.33) whereas the metasedimentary granulites are moderately unradiogenic to chondritic (^87^Sr/^86^Sr_i_ = 0.70458 to 0.70916; ε_Nd_(*t*) = +1.53 to −2.56). In contrast, the Sr-Nd isotopes of Meguma rocks are unradiogenic (^87^Sr/^86^Sr_i_ = 0.7113 to 0.7177; ε_Nd_(*t*) = −8.8 to −11.3) and their middle Proterozoic Nd model ages (T_CHUR_ = 1358 Ma) are significantly older than the early Cambrian (T_CHUR_ = 544 Ma) ages of the metaigneous granulites^16^. The isotopic data and Nd model ages of the metaigneous granulites are indistinguishable from Avalonia magmatic rocks suggesting the Avalon terrane is the structural basement to Meguma^10,16^.

The metasedimentary granulites are of particular significance because they contain heavy mineral detritus (zircon, apatite, monazite) that can be dated using U-Pb radioisotopic methods^21,22^. Detrital zircon geochronology can not only constrain the provenance and depositional age of the host rock but also provides a record of tectonothermal episodes in the catchment area of their sedimentary basin. This is especially important for the metasedimentary granulites as they have not been correlated to surface rocks. Here we use *in situ* laser ablation inductively coupled plasma mass spectrometry (LA-ICP-MS) to date detrital zircons from metasedimentary xenoliths of the Pope’s Harbour dyke. Our results show that the tectonothermal history of the granulites provides important constraints on the pre-, syn- and post accretionary development of Avalonia and Meguma. Moreover, the new data provide the basis for a robust re-interpretation of one of the most debated issues concerning the development of the Northern Appalachians.

## Results

A total of 297 zircons were analyzed from four (PHD-1 = 96, PHD-2 = 84, PHD-3 = 95, SEDXENO = 22) metasedimentary xenoliths (Online Supplementary Dataset S1). Individual zircon grains range in size from 50 µm to 200 µm, and generally have anhedral (rounded, fragmented) to euhedral (prismatic) textures (Figs S1 to S4). The entire ^206^Pb/^238^U age spectrum ranges from 343 ± 6 Ma (1σ) to 2531 ± 21 Ma (1σ) across all samples but the zircons can be divided into an older group comprised predominantly of igneous (Th/U > 0.1) detrital zircon and a younger group of metamorphic (Th/U < 0.1) zircons^23,24^. The metamorphic zircons can be further sub-divided into three distinct populations.

### Detrital zircons

**Figure 2:**
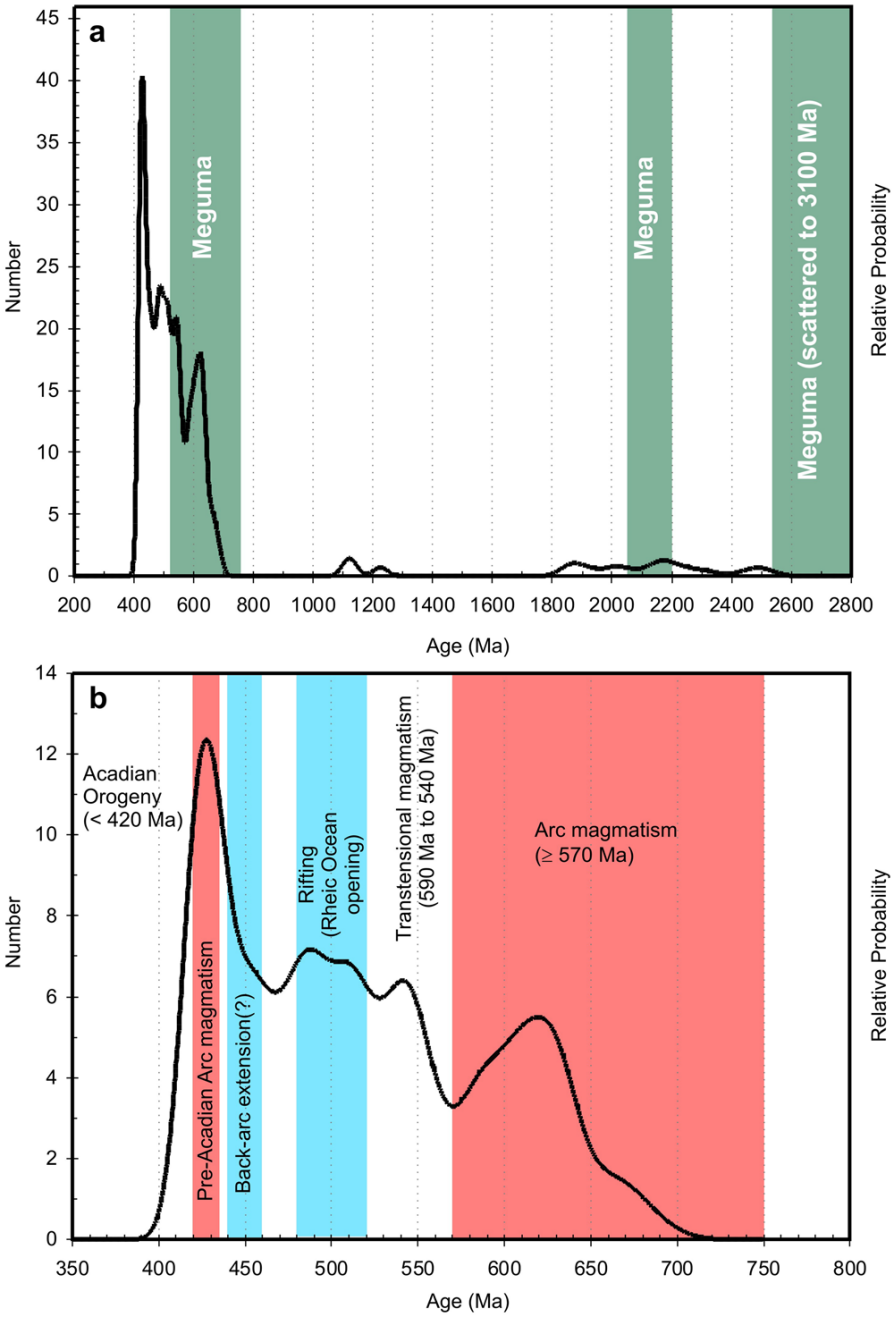
Frequency distribution of detrital zircon ages from the metasedimentary granulite xenoliths of the Pope’s Harbour dyke. (**a)** Total range of detrital zircons ages of the metasedimentary xenoliths compare to the major detrital zircon populations of the Meguma terrane^10^. (**b)** Cryogenian to Early Devonian distribution of detrital zircons showing the major tectonothermal episodes of Avalonia^36^.

The largest group (130) is comprised of zircons with ages ≥415 Ma (415 ± 9 Ma to 2513 ± 43 Ma) although sample PHD-3 did not yield zircons older than 585 ± 13 Ma. The zircons of this group yielded a high average Th/U ratio (0.32 ± 0.5; 2σ) that is typical of an igneous origin. The Proterozoic zircons are comprised of two minor populations with a relatively prominent (12 zircons) Paleoproterozoic group (^207^Pb/^206^Pb ages = 1981 ± 18 Ma to 2618 ± 63 Ma) and a smaller (3 zircons) Mesoproterozoic (^206^Pb/^238^U ages = 1116 ± 24 Ma to 1225 ± 24 Ma) group (Fig. 2a). The remaining zircons (~90%) are Cryogenian to Late Silurian with clusters at 680–660 Ma, ~630 Ma, ~600 Ma, ~580 Ma, 550–450, and 430–420 Ma (Fig. 2b).

### Metamorphic zircons

**Figure 3:**
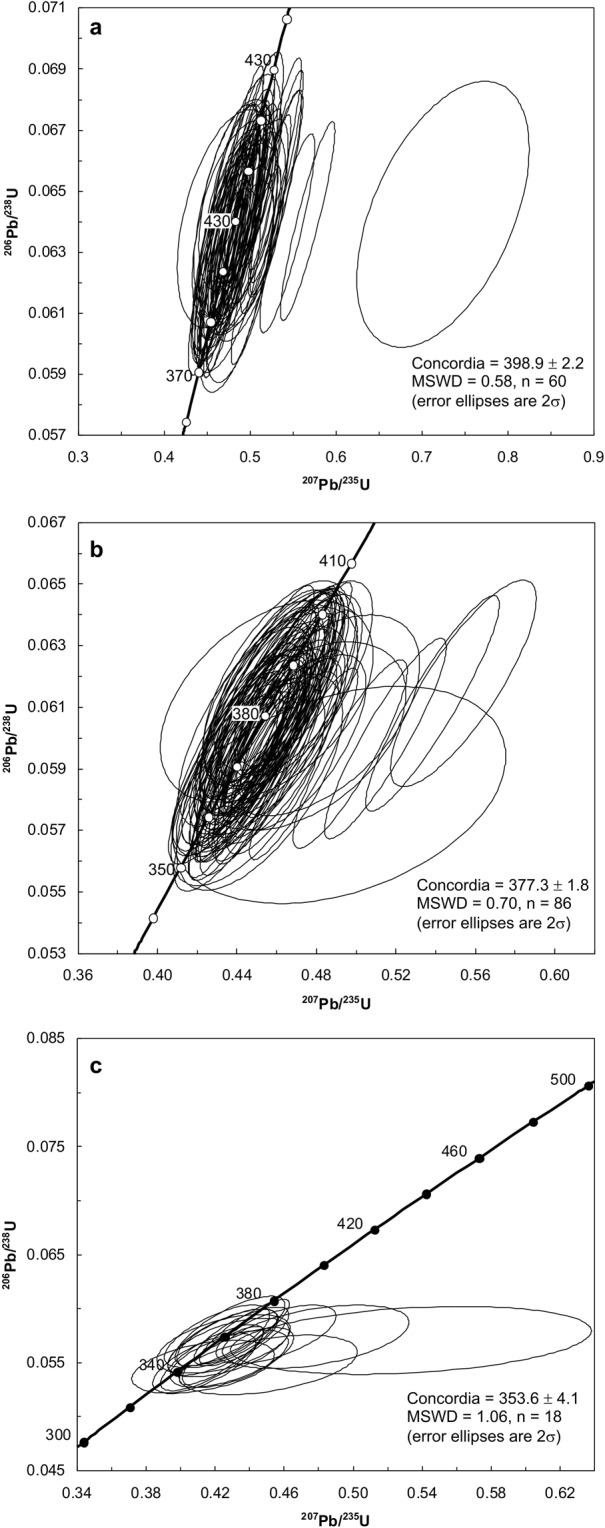
Concordia plots of the metamorphic zircons identified within metasedimentary granulite xenoliths of the Pope’s Harbour dyke. (**a)** Early Carboniferous metamorphic zircons. (**b)** Late Devonian metamorphic. (**c**) Early Devonian metamorphic zircons. Concordia plots of the metamorphic zircons identified within metasedimentary granulite xenolith.

The youngest (<415 Ma) zircons of this study can be separated into three distinct groups. The oldest sub-group comprise 60 zircons and range from 388 ± 8 Ma to 412 ± 9 Ma with a weighted-mean ^206^Pb/^238^U age of 399.0 ± 2.1 Ma (MSWD = 0.59; 2σ) which is indistinguishable from the Concordia (398.9 ± 2.2 Ma, MSWD = 0.58; 2σ) intercept age (Fig. 3a). The average Th/U ratio of this group is 0.05 ± 0.01 (2σ). The middle sub-group consists of 86 zircons that range from 364 ± 9 Ma to 387 ± 8 Ma and produced a weighted-mean ^206^Pb/^238^U age of 376.9 ± 1.6 Ma (MSWD = 0.69; 2σ) and Concordia intercept age of 377.3 ± 1.8 Ma (Fig. 3b; MSWD = 0.70; 2σ). The average Th/U ratio of the middle sub-group is 0.03 ± 0.01 (2σ). The youngest sub-group is comprised of 18 zircons that range from 343 ± 6 Ma to 363 ± 8 Ma and yields a weighted-mean ^206^Pb/^238^U age of 353.8 ± 3.3 Ma (MSWD = 1.00; 2σ) that is within error of the Concordia (353.6 ± 4.1 Ma, MSWD = 1.06; 2σ) intercept age (Fig. 3c). The average Th/U ratio of the youngest zircons is 0.06 ± 0.03 (2σ).

## Discussion

### Provenance and depositional age of the Metasedimentary Granulite

The frequency distribution of the detrital zircon ages from the metasedimentary granulites can help to constrain their provenance. Specifically, the ages can confirm whether the rocks are indicative of Avalonia or Meguma sedimentary rocks. The pre-Neoproterozoic detrital zircons are confined to two groups. The Paleoproterozoic zircons (12) are more abundant than the Mesoproterozoic zircons (3) but they have a diffuse age range (^207^Pb/^206^Pb ages = 1981 ± 18 Ma to 2618 ± 63 Ma). Half of the Paleoproterozoic zircons are ~2.3 Ga (^207^Pb/^206^Pb ages = 2292 ± 17 Ma to 2407 ± 20 Ma) whereas three are ~2.5 Ga (^207^Pb/^206^Pb ages = 2520 ± 16 Ma to 2545 ± 63 Ma). The age range of the Paleoproterozoic zircons overlaps with the ages reported for Meguma sedimentary rocks as well as Avalonia sedimentary rocks (Fig. 2a)^5,11,12,25^. The presence of Mesoproterozoic zircons (^206^Pb/^238^U ages = 1116 ± 24 Ma to 1225 ± 24 Ma), although a small population, is more consistent with an Avalonian provenance as this age is uncommon in detrital zircon studies of Meguma sedimentary rocks^5,11,12,25^.

The post-Mesoproterozoic detrital zircons (≤800 Ma) are the most abundant group and display similar populations identified in Cambrian to Ordovician Avalonia sedimentary sequences from eastern Newfoundland (Avalon, Bonavista, and Burin Peninsulas), the Arisaig and McDonalds Brook Groups of northern Nova Scotia, Mira terrane of eastern Cape Breton and the Broad River Group of southern New Brunswick^25–33^. Moreover, there are Late Silurian (430 Ma to 420 Ma) magmatic zircons that likely represent a contribution of pre- and syn-Laurentia collisional magmatism^25,34^. In contrast, the youngest detrital zircon from the Meguma Supergroup, which the Pope’s Harbour dyke intrudes, is 529 ± 19 Ma and the age of the uppermost formation (Hellgate Falls Formation) is considered to be Floian (477.7 Ma to 470.0 Ma)^12,35^. Thus, the new results provide additional support for the Avalonia affinity of the metasedimentary granulites^16^.

The depositional age of the metasedimentary protoliths is interpreted to be Pridolian as the youngest zircons with high Th/U ratios are ~420 Ma. The Late Silurian zircons are likely derived from some of the last regional magmatic episodes prior to the emplacement of the Middle Devonian granitic batholiths that characterize the main stage of the Acadian Orogeny in the Meguma terrane^1,3,25,34,36^. Therefore the metasedimentary protoliths were amongst the final pre-Acadian Orogeny sedimentary rocks to be deposited on Avalonia. It is possible that the metasedimentary granulites could be the metamorphic equivalents of Arisaig Group (Antigonish Highlands) sedimentary rocks, specifically the Stonehouse Formation (siltstone and shale) as it is considered to be Pridolian (423 ± 1.5 Ma to 419.2 ± 2.8 Ma), comprised of pelitic rocks, and have similar detrital zircon populations^17,25,37^.

### Pre-Acadian Orogeny tectonomagmatic events

It is suggested that proto-Avalonia developed during the Mesoproterozoic as Sm-Nd depleted mantle model ages of Neoproterozoic basement rocks indicates they were derived from a 1.3 Ga to 0.8 Ga tectonothermal event^10,36^. The identification of Mesoproterozoic zircons in this study is consistent with a Mesoproterozoic origin of Avalonia but the provenance of the Paleoproterozoic zircons is less certain as rocks of this age are not found in Avalonia. However, it is thought that proto-Avalonia may have formed along the NW margin of Gondwana and bordered Baltica and/or Amazonia as they have rocks that contain zircons of similar ages as those reported here^6,10,36,38^. More recent studies suggest that detrital zircon Hf isotopes and U-Pb age distributions from Avalonia sedimentary rocks indicate a greater affinity with Baltica specifically, that Avalonia formed as a New Zealand-like ‘ribbon-continent’ that detached from Baltica and accreted to Amazonia by ~670 Ma^39^. Therefore, it appears that the Paleoproterozoic zircons identified in this study are evidence of an ancient cratonic influence during the creation of proto-Avalonia.

**Figure 4:**
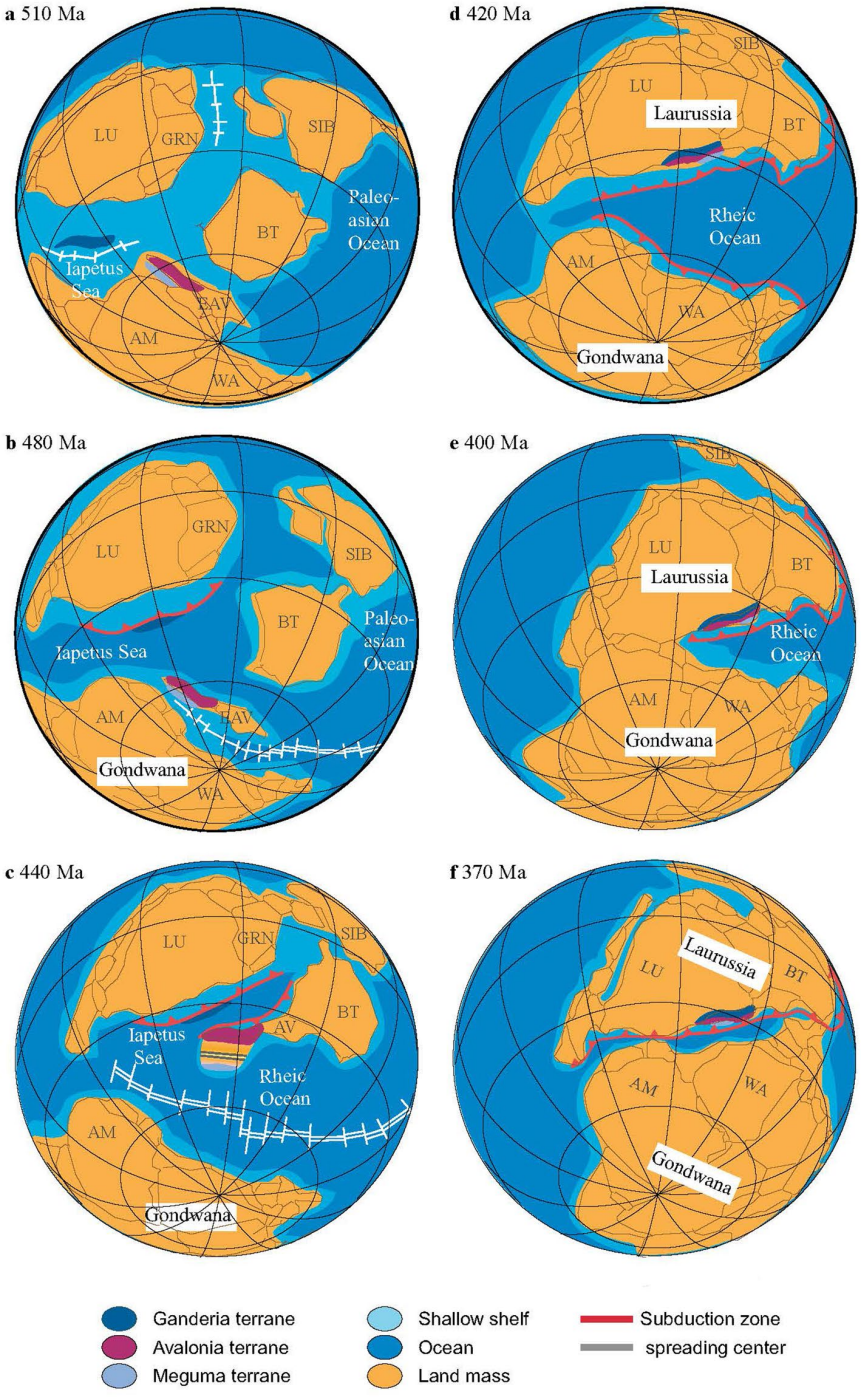
Palinspastic reconstructions during Early Palaeozoic time (510–370 Ma) period. The model uses the tropical latitudes of Laurentia, Avalonia, Siberia, Baltica, and minor terranes which later formed Laurussia from previous models^76^. The evolutionary history of Ganderia (dark blue), Avalonia (violet), and Meguma (purple) terrane along with the evolution of Laurussia and Gondwana are confined with current study. AM = Amazon, AV = Avalonia, BT = Baltica, CG = Congo, EAV = Eastern Avalonia, LU = Laurentia, GRN = Greenland, SIB = Siberia. (**a**) The Ganderia, Avalonia, and Meguma terranes are reconstructed to surround the South America craton around 510 Ma following the breaking up of Pannotia. (**b**) The separation of Ganderia terrane from South America craton as the Iapetus Sea opened. The separation of Avalonia, Eastern Avalonia terrane, and Meguma terrane from the edge of Amazon craton as the Rheic Ocean opened at 480 Ma. (**c**) The closure of Iapetus sea and continuous spreading of Rheic ocean bring Ganderia, Avalonia, Eastern Avalonia terrane closer to Laurentia, but reginal extension between Avalonia and Meguma occurred at 440 Ma. (**d**) Formation of Laurussia during 420 Ma as the Ganderia, Avalonia, and Meguma terranes amalgamated into Laurentia, and the Eastern Avalonia terrane amalgamated into Baltica. (**e**) Further closing of Rheic Ocean around 420 Ma caused the Meguma terrane to be thrust over Avalonia. (**f**) The closure of Rheic Ocean brought Laurussia and Gondwana closer to form the supercontinent Pangea around 370 Ma. Based on our reconstruction, Eastern Avalonia is the only terrane that is close to the West Africa craton during the Early Palaeozoic time period. Although the Carolinian terrane also originated from the edge of West Africa, the rifting and amalgamation of Carolinian terrane occurred during the Late Mississippian, which is much later than for Avalonia.

The younger zircon populations at 680–660 Ma, ~630 Ma, ~600 Ma, 580–530 Ma, 520–450 Ma, and 430–420 Ma correlate to distinct magmatic periods that are identified in western Avalonia and more broadly to the entirety of Avalonia (Fig. 2b). The magmatic ‘pulses’ correspond to: (1) Neoproterozoic early (680 Ma to 670 Ma) and main (635 Ma to 570 Ma) arc-related magmatism; (2) intracontinental transtensional magmatism (590 Ma to 540 Ma); (3) rift-related magmatism (520 Ma to 480 Ma) and Rheic Ocean opening; (4) within-plate (back-arc basin?) related magmatism (460 Ma to 440 Ma); and (5) pre-collision arc-related magmatism from 440 Ma to 425 Ma (Fig. 4)^4,13,27,33,34,36,40–46^. Regional magmatism likely ended by ~420 Ma as there is a dearth of Lochkovian zircons (417 ± 9 Ma to 411 ± 9 Ma) in the metasedimentary granulites and Arisaig Group sedimentary rocks compared to the Pridolian (430 Ma to 420 Ma) and Emsian (407 ± 2.8 Ma to 397.5 ± 2.7 Ma) populations. Late Silurian magmatism is followed by the earliest effects of regional deformation within the Arisaig Group and Meguma Supergroup by 415–410 Ma^14,25,47–50^. Thus there is at least a 5 to 10 million year gap between the cessation of regional subduction-related magmatism and the earliest indication of deformation.

### Syn- and post-Acadian Orogeny metamorphism

Previous studies on the metapelitic xenoliths from the Pope’s Harbour dyke identified three metamorphic episodes^17^. The first (M1) is interpreted to predate the host dyke, and corresponds to a regional, fabric-forming, granulite-facies event. The second (M2) produced undersaturated mineral assemblages (corundum, spinel, sapphirine) that occur within melt blebs that embay biotite and more aluminous M1 phases. Unlike M1 garnets in the metapelite, which have flat compositional profiles typical of granulites, M2 garnet overgrowth rims locally preserve prograde zoning patterns that provide evidence for decompression (i.e.,  rimward decrease in grossular), despite the high metamorphic grade of this assemblage. This testifies to the rapid quenching of the M2 assemblage, consistent with the skeletal character of some M2 phases, notably spinel, the occurrence of ternary feldspar, and the rare preservation of a corundum-quartz paragenesis. These observations suggest a possible syn-emplacement age for M2. Compositional (e.g., mineral zoning) and textural (e.g., overprinting relations) criteria show that the metamorphic mineral assemblages in the xenoliths are in disequilibrium. Thus, the application of mineral thermobarometers to these rocks is challenging. Assuming domainal-scale equilibration between neighbouring minerals, mineral thermobarometry indicates P-T conditions for M1 in the order of 4.5–6.0 kbar at >600 °C. The sapphirine-spinel thermometer suggests that M2 temperatures approached 800 °C^51^. M3 is a greenschist-facies overprint that in some samples obscures M1 and M2 assemblages. The three metamorphic episodes outlined above correspond to regional deformation events that affected the Meguma terrane during the Acadian Orogeny and appear to correlate with the three subgroups of metamorphic zircons^17,47,49,52^.

The oldest group of metamorphic zircons yielded a weighted-mean age of 399.0 ± 2.1 Ma and represents the ‘pre-dyke’ M1 granulite-facies episode. The M1 age is contemporaneous with Early Devonian (~400 Ma) regional greenschist facies deformation within the Meguma terrane^1,14,49^. There is evidence of older deformation (~415 Ma) within Meguma terrane sedimentary rocks^50^ but mica and whole rock ^40^Ar/^39^Ar ages of Halifax Group and Rockville Notch Group (White Rock Formation) rocks yield ages of ~400 Ma that are interpreted to be due to resetting associated with Acadian Orogeny deformation^11,27,53–55^. Furthermore, Emsian to Eifelian (407.6 Ma to 387.7 Ma) zircons are identified in the Brenton pluton and the youngest (Torbrook Formation) sedimentary rocks of the Rockville Notch Group^13,27^. Therefore, the M1 episode likely corresponds to the same deformation event that created the granulite facies conditions experienced by the protoliths of the metasedimentary granulites as well as the greenschist facies conditions of the Meguma Supergroup sedimentary rocks. The most plausible explanation for the contemporaneity of the deformation in both terranes with different conditions is thrusting of the Meguma terrane over Avalonia as this could create the P-T conditions estimated for the granulites without inducing similar conditions to the Meguma Supergroup rocks^17^.

The middle zircon group yielded a Frasnian weighted-mean age of 376.9 ± 1.6 Ma that is within error of previous zircon and monzonite U/Pb TIMS dating (378 ± 1 Ma) of the metasedimentary xenoliths^18^. The Late Devonian age is equivalent to the ‘syn-plutonic’ M2 episode which is contemporaneous with widespread silicic plutonism in the Meguma terrane^7^. The spatially associated Musquodoboit Batholith (800 km^2^) was emplaced at 377.5 ± 0.6 Ma (zircon and monazite U/Pb TIMS) and its estimated crystallization temperature is >650 °C to 800 °C^56^. It is likely that the emplacement of the Musquodoboit Batholith is responsible for the M2 episode as it requires sustained and areally extensive heating to generate melts of sufficient volume. Although the Pope’s Harbour dyke has not been dated, correlative dykes yielded hornblende ^40^Ar/^39^Ar ages of 370 ± 2 Ma and 367 ± 2 Ma^19^. It is likely that the M2 episode corresponds to regional heating associated with the Musquodoboit Batholith and that the metasedimentary xenoliths were entrained in the dyke magma subsequently.

The youngest and also the least abundant metamorphic zircon group yielded a weighted-mean age of 353.8 ± 3.3 Ma which we interpret as the equivalent to the M3 episode. The Early Carboniferous age is contemporaneous with mafic and silicic plutonism in the Cobequid Highlands^8^ although there are no spatially associated intrusions or volcanic rocks near the Pope’s Harbour dyke. However, the greenschist facies overprint is probably associated with the rapid exhumation of the Middle to Late Devonian S-type granites of the Meguma terrane and the oldest movement along the Minas fault zone^1,9,52,57^.

### The Lithotectonic Relationship between Avalonia and Meguma

The lithotectonic relationship between Avalonia and Meguma prior to collision with Laurentia is debated^11,13,14^. On the one hand Meguma is thought to have been contiguous with Avalonia whereas on the other hand it is considered to be an unrelated or at least a distal terrane that was deposited in a rift basin between Gondwana (West Africa or Amazonia) and Avalonia^2,11–13^. One of the most important issues for understanding the pre-accretion relationship between Avalonia and Meguma is the origin of the Rockville Notch Group.

Unconformably overlying the Meguma Supergroup is the Ordovician to Silurian (450 Ma to 420 Ma) Rockville Notch Group, which is comprised of rifted-related (within-plate) volcanic and plutonic rocks (White Rock Formation) and siliciclastic (Torbrook Formation) rocks^2,13,42,53,58^. The volcanic and sedimentary rocks are thought to be either related to the separation of the Meguma terrane from West Africa^13,53,59^ or an overstep sequence^11,27,42,60^. The former interpretation implies there is no pre-collision lithotectonic relationship between Avalonia and the Meguma terrane whereas the latter interpretation indicates they may be contiguous and transited the Rheic Ocean together. Detrital zircon ages and isotopic compositions of Meguma Supergroup rocks are distinct from Avalonian rocks due the comparative absence of Mesoproterozoic (1000 Ma to 1600 Ma) and Ordovician to Silurian zircons^11–13,25,28,59^. However, the Rockville Notch Group has a detrital zircon age distribution that is indistinguishable from the Arisaig Group and the rocks of this study^13,25^. Moreover, the Nd isotopes and depleted mantle model ages of the volcanic and plutonic rocks of the Rockville Notch Group are the same as Avalonian magmatic rocks but different from Meguma^7,10,12,27,43,44,53,61,62^. The implication is that the Rockville Notch Group demonstrates greater affinity with Avalonian rocks than Meguma rocks.

There are three key issues that suggest the Rockville Notch Group is an overstep sequence of Meguma. Firstly, the location of the rift-related volcanic rocks is inconsistent with Meguma separation from Gondwana. Meguma rocks were deposited to the west of Gondwana and therefore the rifted margin, including the volcanic and intrusive rocks (i.e. Rockville Notch Group) should be on the eastern side of the terrane rather than their current location on the western side. There are three scenarios that could explain the current location of the Rockville Notch Group: (1) the Meguma terrane rotated ~180° after separation from Gondwana, (2) the Rockville Notch Group was transported from eastern Meguma to western Meguma during the Acadian Orogeny (~415 Ma) or (3) the Rockville Notch Group is a passive rift sequence within an amalgamated Avalonia-Meguma terrane (i.e. an overstep sequence). At the moment there is little to no evidence to suggest that Meguma rotated 180° during its migration across the Rheic Ocean. Moreover, the contact between the uppermost Halifax Group and lowermost Rockville Notch Group is an unconformity rather than a thrust fault which supports a depositional relationship rather than a tectonic relationship^58,63^.

Secondly, the ~440 Ma Brenton pluton, a subvolcanic intrusion correlative with the White Rock Formation volcanic rocks, contains a significant population of inherited zircons with ^206^Pb/^238^U ages between 460 ± 11 Ma to 499 Ma ± 12 Ma including a cluster between 472 ± 11 Ma and 485 Ma ± 12 Ma^13,27^. The implication is that the inherited zircons must be derived from older (>440 Ma) lithologies that contain Early to Middle Ordovician zircons. Early to Middle Ordovician detrital minerals are absent in the Meguma Supergroup^5,12,64,65^. Thus, either the Meguma terrane had rocks with Early to Middle Ordovician zircons that were removed sometime after the emplacement of the Brenton pluton but before collision with Avalonia or, the magmatic rocks of the Rockville Notch Group were generated from an Avalonian lithospheric source that subsequently erupted on top of, and possibly through, the Meguma Supergroup.

Thirdly, the time interval between rifting of the Meguma terrane from Gondwana and its collision to Laurentia may be too short given the distance. The youngest rift-related magmatic rock of the Rockville Notch Group is the Late Silurian (426 ± 2 Ma) Mavillette gabbro^66,67^. If rifting from Gondwana began at ~440 Ma and magmatism ceased by ~425 Ma, then the Meguma terrane crossed the Rheic Ocean - a distance estimated to be 4000 km - and collided with Avalonia in only 10–20 Ma^68^. This scenario would require the Meguma terrane to rotate 180° after separation from Gondwana and then transit the Rheic Ocean at rate of 20–40 cm/year.

**Figure 5:**
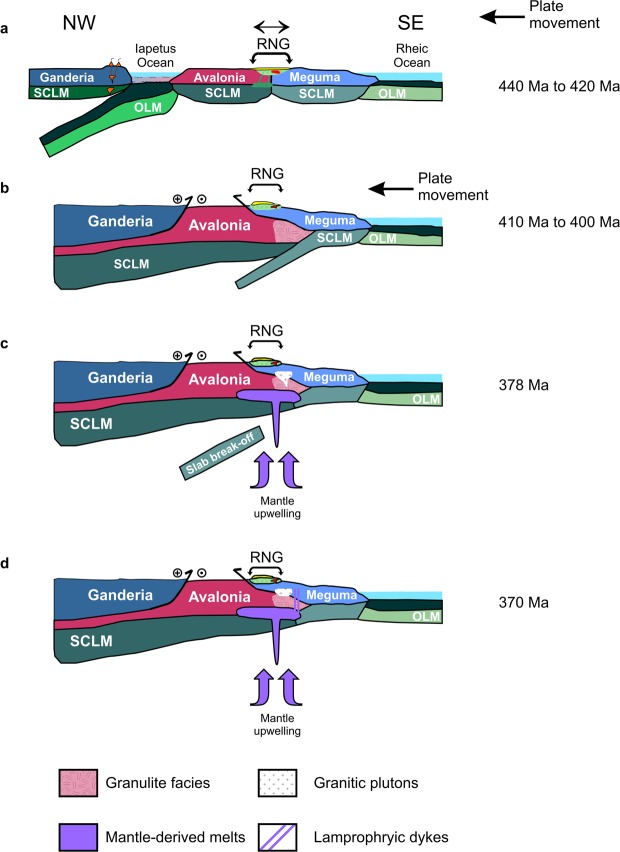
Tectonic evolution showing the possible relationship between Avalonia and Meguma during the Late Silurian to Late Devonian. (**a**) Late Silurian emplacement of the Rockville Notch Group (440 Ma to 420 Ma). (**b**) Initial stages of the Acadian Orogeny and peak granulite facies metamorphism (M1) in the underlying Avalonia rocks (410 Ma to 400 Ma). (**c**) Middle Devonian silicic magmatism (378 Ma) and the high temperature M2 deformation episode. (**d**) Late Devonian dyke emplacement and entrainment of the granulite xenoliths (~370 Ma). SCLM = subcontinental lithospheric mantle. OLM = oceanic lithospheric mantle. RNG = Rockville Notch Group.

Based on the detrital age spectra, Nd isotope systematics, and geological relationships, it is very likely that the Rockville Notch Group is an overstep sequence that has affinities with Avalonia. It is possible that the Rockville Notch Group volcanic rocks are related to ensialic (passive) rift-related magmatism found in the Antigonish Highlands (McGillivray Brook Formation) as they have similar isotopic compositions (ε_Nd_(*t*) = +0.2 to +6.8), depleted mantle (T_DM_ ≈ 1200 Ma) model ages, and broadly similar emplacement ages (McGillivray Brook = 454 ± 0.7 Ma, Rockville Notch = 443 ± 2; 442 ± 4; 438 + 3/−2 Ma)^13,43,53,62^. Figure 5 outlines a possible scenario which may explain the lithotectonic association between Avalonia, Rockville Notch Group, and the Meguma terrane. We suggest that the Rockville Notch volcanic rocks erupted within a rift basin that developed near the boundary between Avalonia and Meguma. The cause of rifting is uncertain but it could be related to tensional stress induced by westward subduction of the Iapetus Ocean just prior to the collision between Laurentia and Avalonia (~420 Ma to ~410 Ma) or to a combination of tensional plate stresses related to Iapetan subduction and Rheic extension. Consequently, the most plausible lithotectonic relationship between the Avalonia and Meguma terranes since the opening of the Rheic Ocean is one of contiguity and that they transited together.

## Conclusions

The geochronology results of this study show that the majority of detrital zircons have ages between ~420 Ma and ~660 Ma with minor groups at ~1100 Ma and ~2200 Ma. The new results are supportive of an Avalonia provenance of the metasedimentary granulites with possible influence of pre- to syn-Laurentia collision-related magmatic rocks during the Late Silurian. The protoliths depositional ages are interpreted to be ~420 Ma. Younger zircons (<410 Ma) correlate to three distinct tectonomagmatic events: (1) the early period of the Acadian Orogeny (399.0 ± 2.1 Ma), (2) the main period of Acadian Orogeny-related magmatism at 376.9 ± 1.6 Ma, and (3) post-Acadian Orogeny regional exhumation and Minas fault zone movement during the Early Carboniferous (353.8 ± 3.3 Ma). The contrasting nature of the metamorphic conditions experienced by the metasedimentary xenoliths (granulite facies) and Meguma Supergroup (greenschist facies) rocks at the same time can best be explained by collision and thrusting of Meguma over Avalonia at ~400 Ma. The implication is that there was ~20 million years between the deposition of the protolith and peak metamorphic conditions. Consequently, the Meguma terrane was proximal to Avalonia prior to collision and very likely contiguous.

## Methods

### Laser ablation inductively coupled plasma mass spectrometry (LA-ICP-MS) zircon geochronology

Four metasedimentary xenoliths (2–4 kg each) were collected from the Popes Harbour dyke, Nova Scotia located at 44°46′47.55″N, 62°38′56.88″W. Zircons were separated using magnetic separation and heavy-liquid techniques at the Yu-Neng Rock and Mineral Separation Company (Lanfang, Hebei). Cathodoluminescence (CL) images were taken at the Institute of Earth Sciences, Academia Sinica, Taipei for the examination of the individual crystal internal structures and for selecting suitable positions for U-Pb analyses. Zircon U-Pb isotopic analyses were performed by laser ablation-inductively coupled plasma mass spectrometry (LA-ICP-MS) at the Department of Geosciences, National Taiwan University, Taipei using an Agilent 7500 s Q-ICP-MS and a Photon Machines Analyte G2 193 nm laser ablation system. A spot size of 35 μm with laser repetition rate of 5 Hz was used and the laser energy density was 3.83 to 5.33 J/cm^2^. Calibration was performed by using the zircon standard GJ-1 (608.5 ± 0.4 Ma)^69^, 91500 (1065 Ma)^70^, and zircon Plešovice (337.1 ± 0.4 Ma)^71^ was also used for data quality control. Measured U-Th-Pb isotope ratios were calculated using the GLITTER 4.4.4 software^72^ and the relative standard deviations of reference values for GJ-1 were set at 2%. The common lead was corrected using a common lead correction function^73^, and the weighted mean U-Pb ages, Concordia plots and probability density plots were created using Isoplot v. 4.1^74^.

### Plate reconstruction

The reconstructions were created using the open-source software GPlates 2.1 following the model suggested by the Paleomap project database^75^ provided within: https://www.earthbyte.org/.

## Supplementary information

Figures S1-S4

Dataset 1

## Data Availability

The authors declare that all analytical data supporting the findings of this study are available within the paper and its Supplementary Information Files.
