# Supplementary material for: Long-lived association between Avalonia and the Meguma terrane deduced from zircon geochronology of metasedimentary granulites
Source: Sci Rep. 2019 Mar 11;9:4065. doi: 10.1038/s41598-019-40673-9 (PMC6411879; doi:10.1038/s41598-019-40673-9)
Supplement: Supplementary file 1 — Figures S1-S4 [file 41598_2019_40673_MOESM1_ESM.doc]

# Supplementary Figures

# Long-lived association between Avalonia and the Meguma terrane deduced from zircon geochronology of metasedimentary granulites

J. Gregory Shellnutt^1^, J. Victor Owen^2^, Meng-Wan Yeh^1,3^, Jaroslav Dostal^2^ & Dieu T. Nguyen^1^

*^1^National Taiwan Normal University, Department of Earth Sciences, 88 Tingzhou Road Section 4, Taipei 116, Taiwan*

*^2^Saint Mary’s University Department of Geology, 923 Robie Street, Halifax, NS, B3H 3C3, Canada*

*^3^Center for General Education, National Taiwan Normal University, 162 Heping East Road Section 1, Taipei 106, Taiwan*

*Correspondence and requests for materials should be addressed to J.G.S. (email:jgshelln@ntnu.edu.tw)

**Supplementary Figures**


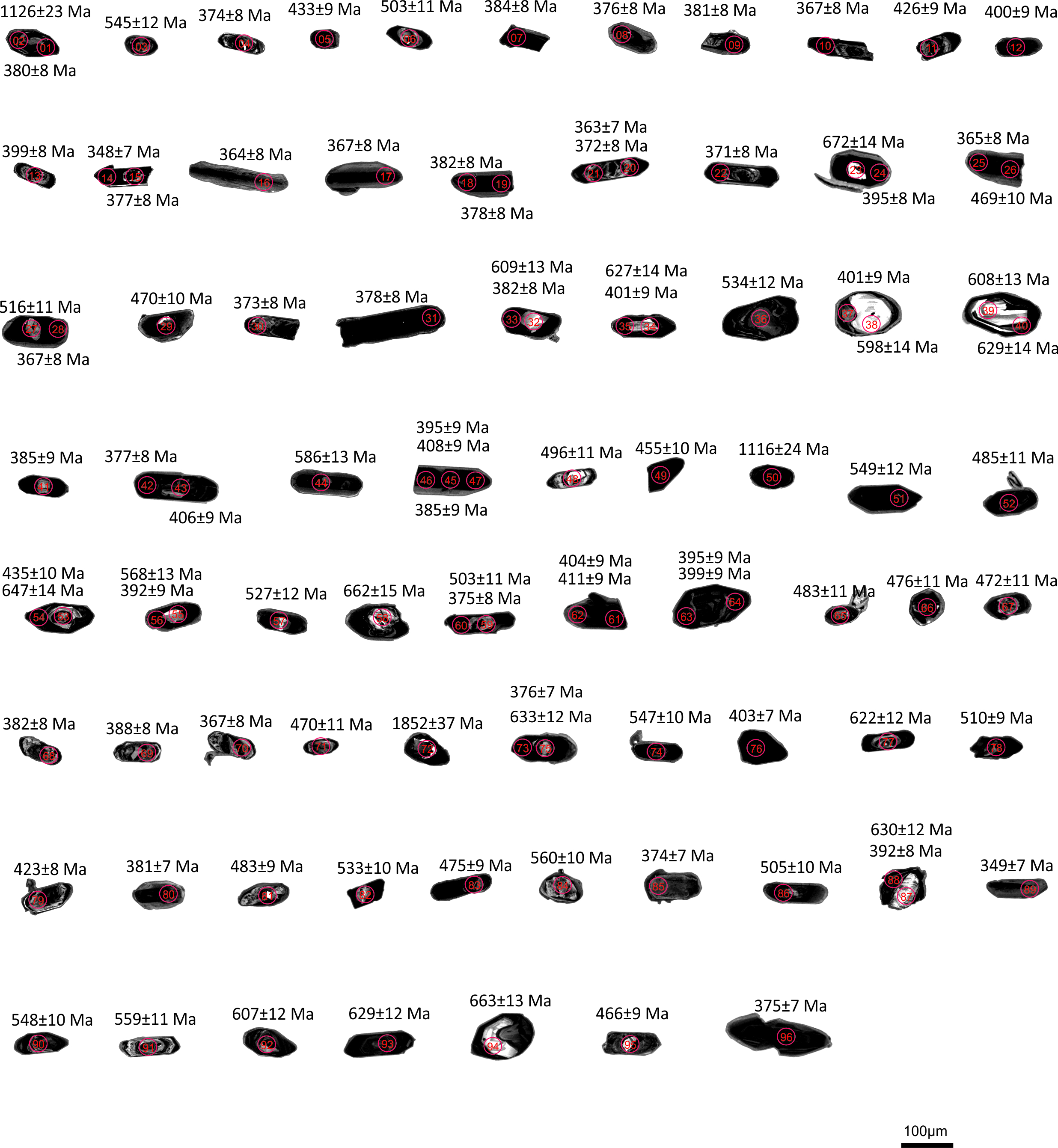


**Fig. S1** Cathodoluminescence images of the zircons from sample PHD-1.

**
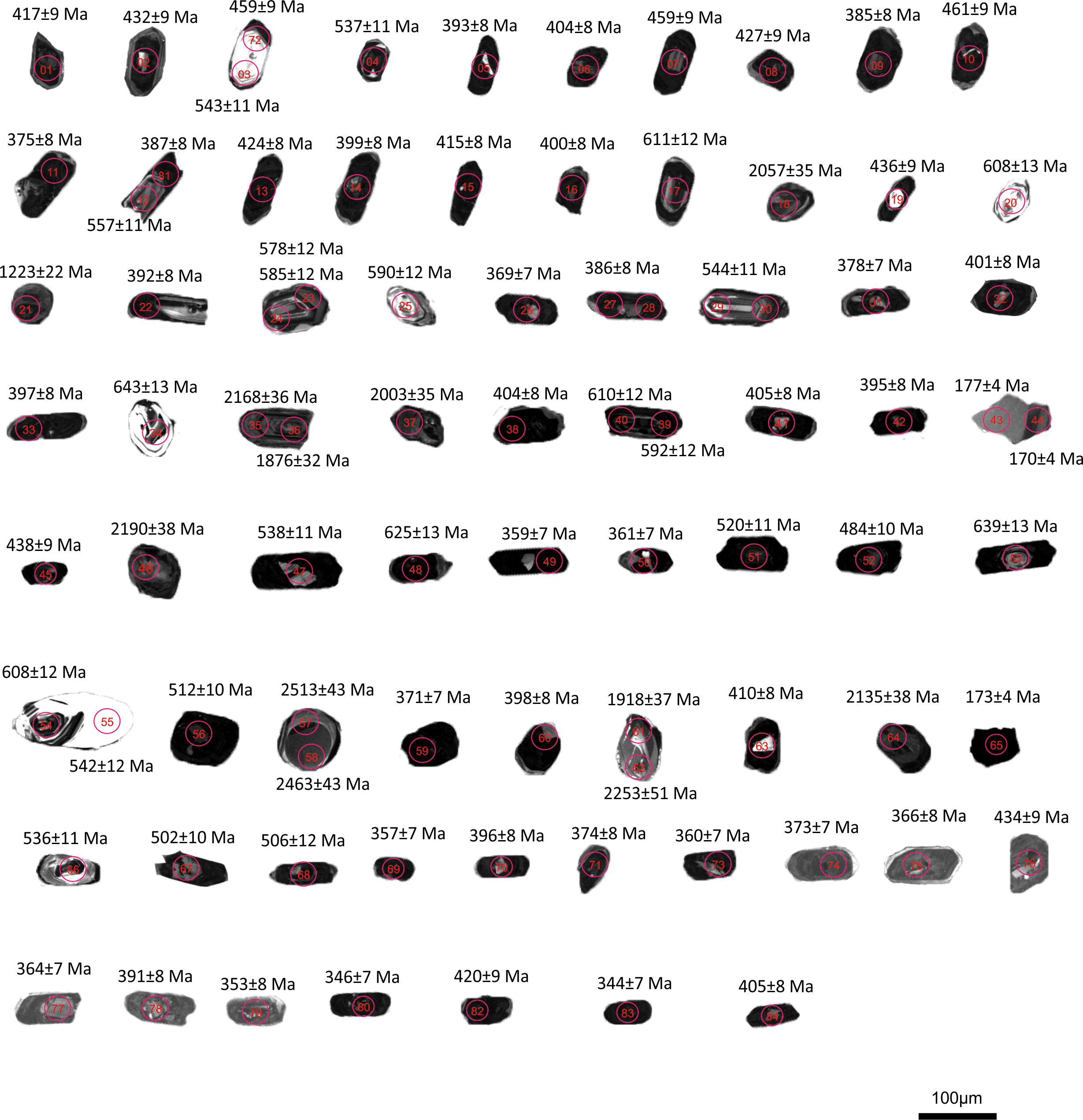
**

**Fig. S2** Cathodoluminescence images of the zircons from sample PHD-2.


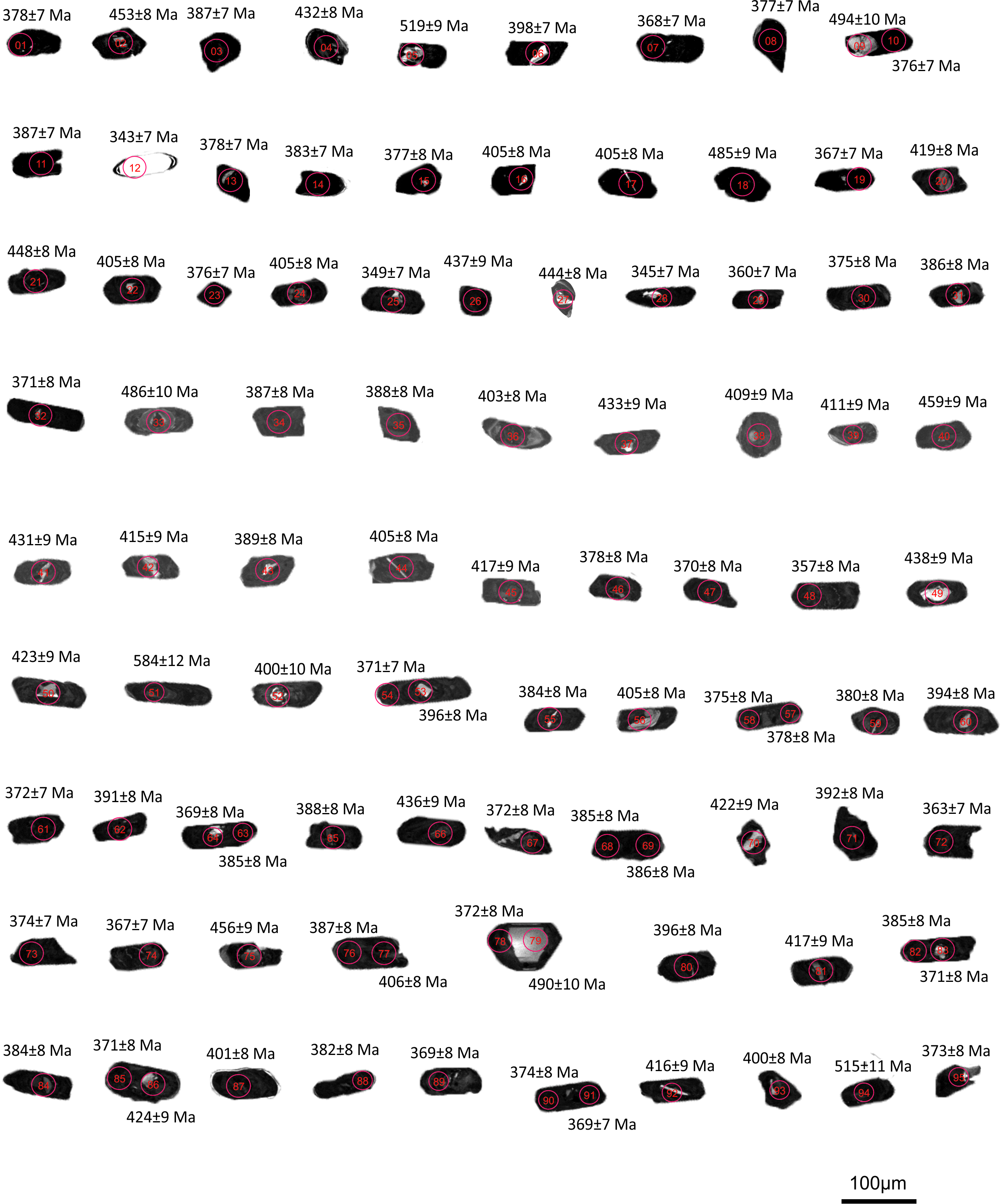


**Fig. S3** Cathodoluminescence images of the zircons from sample PHD-3.


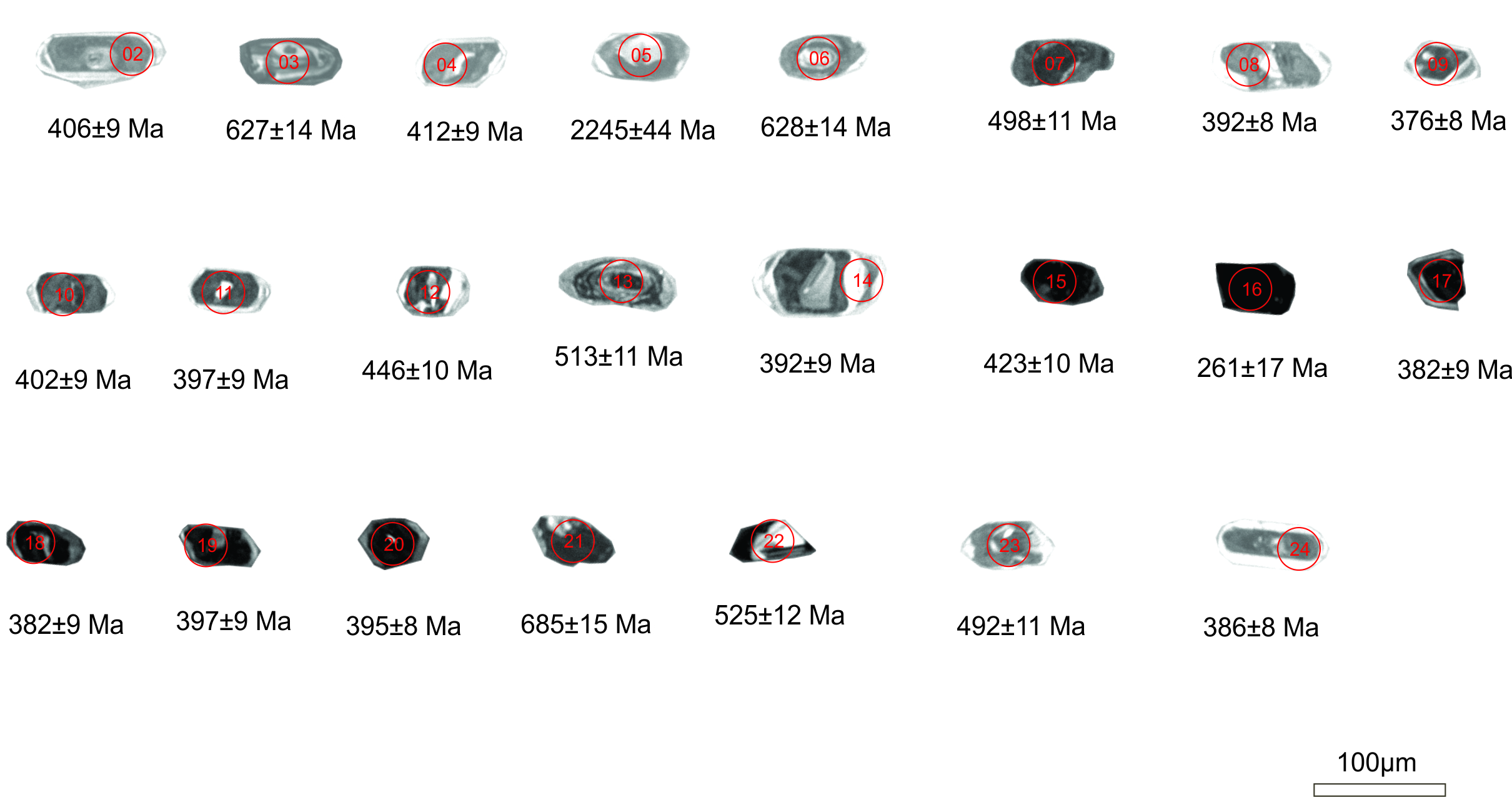


**Fig. S4** Cathodoluminescence images of the zircons from sample SEDXENO.
